# Evaluation of Dried Sweet Sorghum Stalks as Raw Material for Methane Production

**DOI:** 10.1155/2014/731731

**Published:** 2014-08-19

**Authors:** Leonidas Matsakas, Ulrika Rova, Paul Christakopoulos

**Affiliations:** Biochemical Process Engineering, Division of Chemical Engineering, Department of Civil, Environmental and Natural Resources Engineering, Luleå University of Technology, 971-87 Luleå, Sweden

## Abstract

The potential of utilizing dried sweet sorghum stalks as raw material for anaerobic digestion has been evaluated. Two different treatments were tested, a mild thermal and an enzymatic, alone or in combination. Thermal pretreatment was found to decrease the methane yields, whereas one-step enzymatic treatment resulted in a significant increase of 15.1% comparing to the untreated sweet sorghum. Subsequently, in order to increase the total methane production, the combined effect of enzyme load and I/S on methane yields from sweet sorghum was evaluated by employing response surface methodology. The obtained model showed that the maximum methane yield that could be achieved is 296 mL CH_4_/g VS at I/S ratio of 0.35 with the addition of 11.12 FPU/g sweet sorghum.

## 1. Introduction

Replacement of fossil fuels with renewable energy carriers is now more urgent than ever in order to minimize the negative impacts of human activities on the environment. Biogas provides a renewable alternative to the traditional fossil fuels and is produced from anaerobic digestion of organic materials [1, 2]. Anaerobic digestion is a complex biochemical process which takes place in the absence of oxygen and involves several steps (hydrolysis, acidogenesis, acetogenesis, and methanogenesis) where each of them is catalyzed by different category of microorganisms and all together form a unique system where the products of one step are the raw materials of the next [3–5]. Taking into account the resource efficiency, biogas production is considered to have a better ratio of output to input energy comparing to ethanol production [6]. Special care should be taken in order not to inhibit the methanogenesis process, as it is more sensitive compared to the other steps [4].

The main components of the biogas are methane (CH_4_) and carbon dioxide (CO_2_) and the ratio between them affects the total energy of biogas which is estimated to vary between 18,630 and 26,081 kJ/m^3^ [7]. Biogas has different applications, as it can be used for electricity and heat production or as vehicle fuel [6]. Another important benefit of biogas production is that the resulted digestate can be utilized as biofertilizer as it presents increased nutrient availability and favorite elemental composition [1, 4, 5].

Different kinds of organic materials have been utilized as raw material for anaerobic digestion, such as sewage, different animal manures, various food residues [8–10]. There is a great interest of exploiting solid biomass for biogas production and more specifically lignocellulosic biomass which is a highly abundant resource and available at low cost [11]. One important and crucial step during anaerobic digestion of lignocellulosic material is the efficient hydrolysis of the complex carbohydrates cellulose and hemicellulose [5, 7]. Improving the efficiency of this step is of great importance in order to achieve high biogas production yields.

Biomass could be derived, for example, from agricultural, such as different straws, or forest residues. An alternative source of biomass could be energy crops like sweet sorghum. Cultivation of sweet sorghum presents several benefits as it requires fewer inputs (like fertilizers) and due to the high photosynthetic activity that presents high amounts of soluble and insoluble carbohydrates are produced in a short period [12–14]. Moreover it is tolerant to harsher climate conditions (like drought and high soil salinity) and can exploit lands that are not suitable for the cultivation of other crops [13, 15]. On the other hand, presence of soluble sugars in stalks results in low storage stability of sweet sorghum, which in turn makes storage of stalks a challenge and all-around year availability of them difficult. This problem can be solved by drying of stalks, as has previously been demonstrated [16, 17].

The aim of this work was to evaluate the potential use of dried sweet sorghum stalks as raw material for the production of biogas using a thermophilic sludge. Utilization of thermophilic conditions rather than mesophilic presents some benefits such as more thermodynamically favorable conditions, leading to higher methanogenic activity and in turn faster digestion, and less contamination problems from other microorganisms [11, 18]. Different treatments of sweet sorghum stalks were also evaluated concerning the improvement of methane production yields.

## 2. Materials and Methods

### 2.1. Feedstock and Inoculum

During this work the Keller variety of sweet sorghum was utilized, which was cultivated in Voiotia region of central Greece. Preparation of dried sweet sorghum stalks was done as previously described [16]. The particle size after drying and milling was 0.75 mm. Volatile solids (VS) concentration of sweet sorghum stalks was 93.44% w/w, whereas total solids (TS) content was 95.69% w/w. The composition of sweet sorghum stalks per dry weight is as follows (% w/w): sucrose, 34.4; glucose, 8.2; fructose, 8.1; cellulose, 19.6; hemicellulose, 15.2; acid insoluble lignin, 3.2 [16].

Anaerobic sludge which was used as inoculum was collected from the biogas plant in Boden, Sweden, where biogas is produced by thermophilic codigestion of sewage sludge and food wastes at 55°C. The VS and TS content of the inoculum were 1.17% w/w and 2.04% w/w, respectively.

### 2.2. Thermal and Enzymatic Treatment

During this work two different treatments were applied to improve methane yields, one thermal and one enzymatic, alone or in combination. Thermal treatment was performed using an autoclave apparatus at 105°C for 1 hour with sweet sorghum's concentration of 20% w/w.

During enzymatic treatment, a mixture of the commercial enzyme solutions Celluclast 1.5 L and Novozym 188 (Novozymes A/S, Denmark) at 5 : 1 v/v ratio was used at the same enzyme loading that was previously found optimal for sweet sorghum saccharification during ethanol production [16]. Two different process configurations were evaluated during the enzymatic treatment, namely, one-step and two-step processes. In one-step process, the enzymes were directly added in the sludge, whereas in the two-step process sweet sorghum was enzymatically presaccharified prior to the addition to sludge. During the two-step process the saccharification was performed at 50°C for 8.6 hours at 20% w/w DM content. In order to avoid the hydrolysis of sucrose by Novozym 188 endogenous invertase activity and subsequent inhibition of cellulases, the enzymatic solution was added in the startup of anaerobic digestion stage.

### 2.3. Analytical Methods

TS content was measured as weight difference before and after drying the samples at 105°C for 24 hours. The VS content was measured after drying the sample at 550°C for 2 hours and abstracted this weight difference from the TS content.

Enzyme activity of the commercial enzyme solutions was measured according to the method developed by Ghose [19] and found to be 83 FPU/mL for the mixture.

### 2.4. Biochemical Methane Potential (BMP)

BMP assays were performed at the Automatic Methane Potential Test System (AMPTS II) of Bioprocess Control AB (Lund, Sweden). Incubation took place in 500 mL glass bottles containing 400 g of total sample (inoculum and substrate). Slow mixing of the sludge was conducted by motors on the top of each flask at intervals of 10 min mixing and 1 min resting. Every bottle was connected with a CO_2_-fixing unit, which consists of 100 mL glass flasks containing approximately 80 mL of 3 M NaOH and thymolphthalein as pH indicator. Finally, the volume of the methane was measured at the gas flow meter unit.

In every batch of experiments two different controls were also included. One with only the inoculum in order to calculate the methane production from the organic load already present in the sludge and one with the inoculum and the enzymes in order to calculate methane production from the digestion of the enzymes. Finally, a positive control experiment was also included to evaluate the quality of the sludge, containing avicel cellulose. During the first batch of experiments the inoculum to substrate ratio (I/S ratio) in terms of VS was equal to 2, whereas in the second batch it varied as described in Section 2.5. Each flask was supplemented with salt and trace element solution as described by Antonopoulou and Lyberatos [12]. Prior to startup of the digestion each flask was sparged with nitrogen for 90 sec. Incubation of the flasks took place in a water bath at 55°C until no significant amounts of methane were produced. All the experiments lasted a maximum of 21 days.

### 2.5. Experimental Design

An experimental design (response surface methodology (RSM)) was employed during this work in order to evaluate the combined effect of enzyme loading and I/S ratio on the methane yield. RSM allows the estimation of the interactions of the chosen factors and their effect in one or more responses and can result in improvement of the process factors. According to the specific experimental design employed, different combinations of the chosen factors, which vary at certain levels, are generated. The responses of these combinations can be graphically represented and a quadratic or cubic model can describe the behavior of the responses. During this work a Box-Wilson circumscribed central composite (CCC) design was employed generating 11 experimental combinations (3 replicates of the central points) which were done in duplicates (Table 1). The quadratic model applied was the following:

(1) Met=β0+β1·X1+β2·X2 −β3·X12−β4·X22−β5·X1·X2,

where met represents the methane yield per gram of VS (mL CH_4_/g VS), *X*_1_ the enzyme loading (FPU/g sweet sorghum), and  *X*_2_ the I/S ratio and with *β*_*i*_ the different coefficients. Fitting of the model according to multiple linear regression (MLR) and statistical analysis of the obtained model was done using the software MODDE v.10 of Umetrics.

## 3. Results and Discussion

### 3.1. Evaluation of Different Treatments on Methane Potentials

Sweet sorghum stalks contain both soluble (glucose, fructose, and sucrose) and insoluble carbohydrates (cellulose and hemicellulose) which could be utilized for methane production. Despite the fact that the methane producing consortia can hydrolyze insoluble carbohydrates, the methane yields could be lower when the lignocellulosic materials are utilized without any kind of treatment [6, 20, 21]. One way to increase digestibility of insoluble carbohydrates is the application of a physicochemical pretreatment process, such as hydrothermal, dilute acid, and steam explosion. On the other hand pretreatment of sugar crops like sweet sorghum, which contain high amounts of soluble sugars, can result in formation of inhibitors (such as furfural and HMF) leading to a significant decrease of the available sugars. For this reason the application of a physicochemical pretreatment at harsh conditions (e.g., high temperature or treatment duration) is not feasible. On the other hand, addition of hydrolytic enzymes could facilitate the hydrolysis of both cellulose and hemicellulose and in turn increase the methane yield.

During this work an enzymatic treatment was evaluated by employing a mixture of Celluclast 1.5 L and Novozyme 188 at a ratio of 5 : 1 volumes, at a concentration equivalent to 8.32 FPU/g sweet sorghum (as previously found optimal for sweet sorghum saccharification by Matsakas and Christakopoulos [16]). In order to evaluate the effect of enzymatic treatment, two different process configurations were evaluated, namely, a one-step and two-step process which resemble the SSF (simultaneous saccharification and fermentation) and SHF (separate saccharification and fermentation) processes during bioethanol production from lignocelluloses.

**Figure 1:**
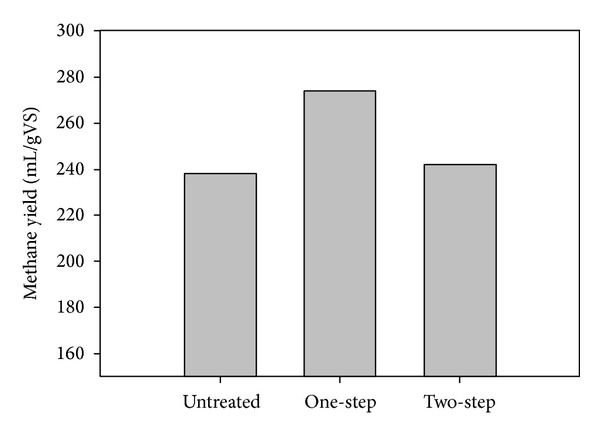
Effect of enzymatic treatment on methane yields. Enzymatic treatment was performed either in one step or in two steps. The values presented are the average of duplicate experiments.

When no treatment was applied to sweet sorghum stalks methane yield reached 238 mL/g VS. In contrast, addition of enzymes improved the overall methane production yields (Figure 1). It is worth noticing that when a two-step process configuration was applied the increase of methane yield was only 1.7%, whereas during the one-step process the increase was 15.1% reaching a methane production of 274 mL/g VS with the most probable reason being the presence of higher initial sugar concentration in the startup of anaerobic digestion stage during the two-step process, which could result in production of higher amounts of volatile fatty acids (VFAs), which in combination with lowering the pH below optimal could have a negative impact on methane production [22, 23].

**Figure 2:**
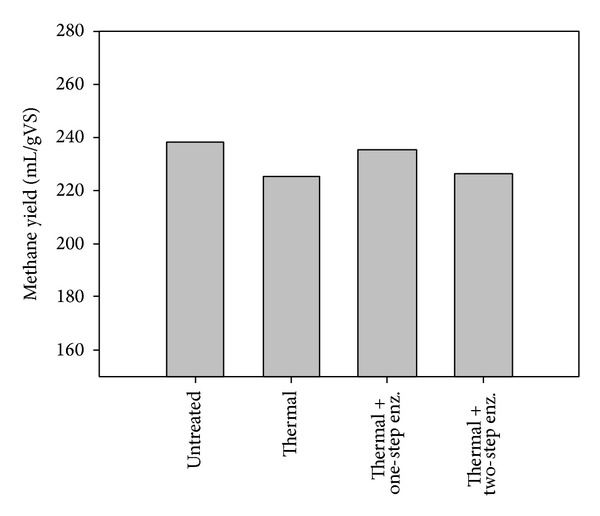
Effect of thermal treatment on methane yield with or without the combination of enzymatic treatment. The values presented are the average of duplicate experiments.

Subsequently the effect of a mild thermal pretreatment (105°C for 1 h) on sweet sorghum digestibility was evaluated without the addition of any acid or basic catalyst which could result in severe degradation of soluble sugars. It was previously reported that a thermal pretreatment under mild conditions could enhance methane production from sweet sorghum stalks [12]. Despite the fact that Antonopoulou and Lyberatos [12] found a positive effect of thermal pretreatment on sorghum digestibility, during this work the methane production of pretreated sweet sorghum was 5.46% less compared to the untreated one, resulting in a methane production of 225 mL/gVS. This could be attributed to the minor degradation of soluble sugars and formation of inhibitors. Addition of enzymes to the thermally pretreated sweet sorghum increased the methane production but the overall yield was less compared to the yield obtained by the untreated sweet sorghum (Figure 2). The same negative effect of the two-step process was also observed during utilization of thermally pretreated sweet sorghum stalks.

### 3.2. Evaluation of the Combined Effect of Enzyme Loading and I/S Ratio on Methane Production

From the previous experiments it was concluded that a one-step enzymatic treatment step increases the methane yields from sweet sorghum. During anaerobic digestion the I/S ratio is considered to play a very important role on the methane yields [2, 24]. If this ratio is low, there is high possibility of inhibition of methane production due to the accumulation of VFAs [1], which is a result of the imbalance between the acidogenic and methanogenic stage [4]. On the other hand, lower I/S ratio results in higher substrate concentrations which in turn yields in higher total methane production per volume of sludge, which is very important for the economic viability of the process. Thus it is important to find the lowest I/S ratio in which the methane yield per gram of volatile solids is not decreasing and at the same time the total production of methane is high.

**Figure 3:**
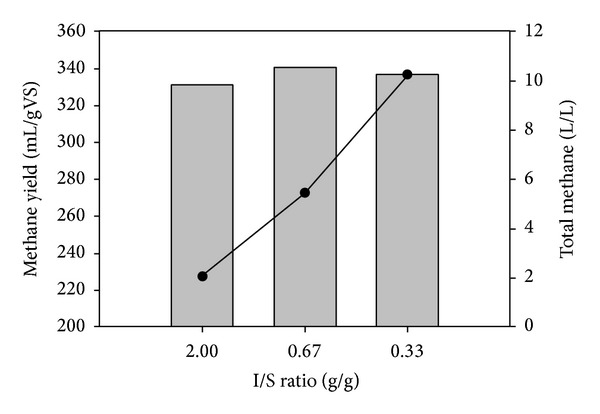
Effect of I/S ratio in methane yield and total methane production from avicel cellulose. The values presented are the average of duplicate experiments.

In order to evaluate the ability of the consortium to act at low I/S ratio, initially microcrystalline cellulose was employed at different I/S ratios (2, 0.67, and 0.33). As can be seen in Figure 3 methane production per gram of VS is increased with decreasing I/S to 0.67, while further decrease to 0.33 resulted in slight decrease of methane potential, which was still above the methane yields at I/S ratio 2. It can be concluded that the used microbial consortium is capable of digesting materials at low I/S ratios which results in higher overall methane production, which in this case increases from 2.1 L CH_2_/L to 10.2 L CH_2_/L when I/S ratio is decreased from 2 to 0.33.

Subsequently the combined effect of enzyme load and I/S ratio was evaluated by response surface methodology according to circumscribed central composite (CCC) design. 11 experimental combinations came from the experimental design as represented in Table 1 which were done in duplicate resulting in a total of 22 experiments. During the initial fitting of the quadratic model to the obtained results it was found that the *R* ^2^ was 0.495, whereas the *Q* ^2^ was −0.067, values that indicate that the model was not adequate enough to describe the experimental values and predict values at new experimental combinations. For this reason the values of the experiments at the combination 8 FPU/g sweet sorghum and 0.16 g/g I/S ratio were excluded, as the methane production was inhibited (Table 2). The obtained model is described by the following equation:

(2) Met=278.708+0.716472·X1−16.9907·X2 +0.014611·X12−1.58786·X22+0.594616·X1·X2.

**Figure 4:**
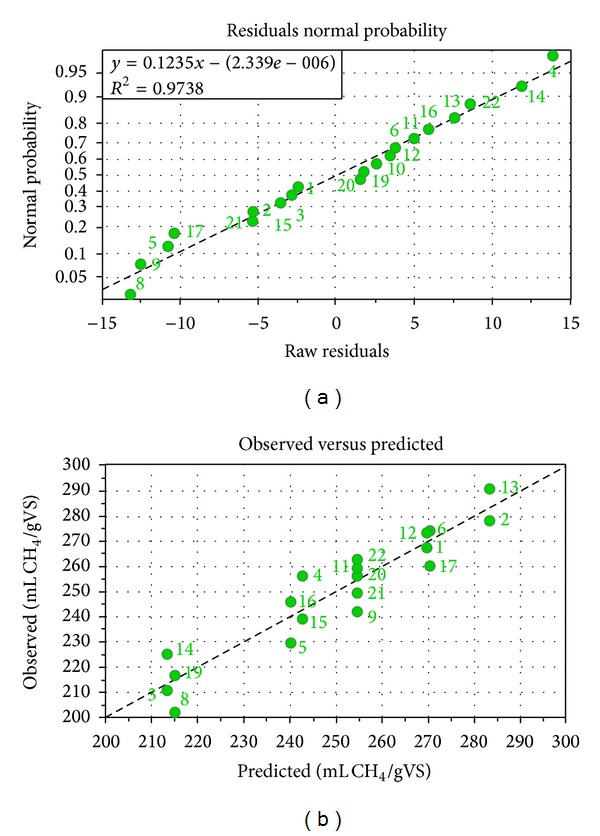
Diagnostic tools for model evaluation. (a) Residual normal probability and (b) plot of observed values against predicted.

*R* ^2^ was improved to 0.886 and the *Q* ^2^ to 0.762 indicating that the model is capable of fitting the experimental data and can efficiently predict new data. Two more factors that describe the efficiency of a model are the model validity and the reproducibility. For the model obtained during this work, both of them were high and found to be 0.735 and 0.849, respectively. Finally, two diagnostics tools were employed to verify the adequacy of the model to fit experimental data, namely, the normal probability plot of residuals and the relationship between predicted and experimental data (Figure 4). Normal probability plot of residuals is made by plotting the observed residuals against the expected values [24] and is used to evaluate the normality of the residuals as well as to detect outliers, whereas plot of experimental obtained data versus predicted ones indicates the efficiency of the model to describe the obtained experimental results. The model obtained during this work is sufficiently describing the experimental result as the values of the data are fairly close to the linear line. This can also be observed at Table 2 where the experimental and predicted values for the duplicate experiments are given.

**Figure 5:**
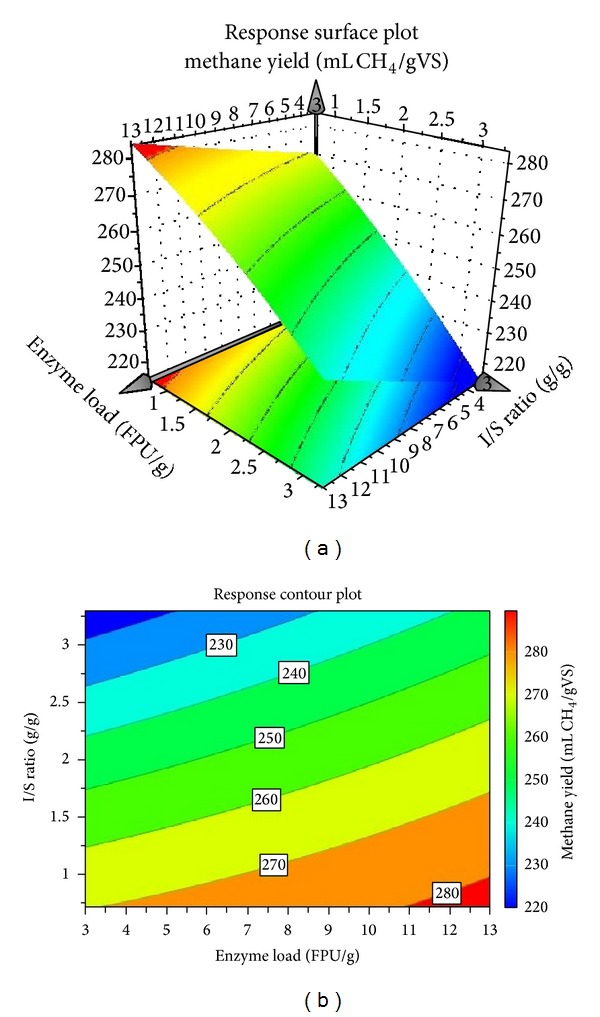
Response surface (a) and contour plot (b) of the methane yield at different combinations of enzyme load and I/S ratio.

Figure 5 shows the resulting response surface and contour of the model. It can be observed that low I/S ratio in combination with higher enzyme loadings lead in increased methane yields, where the yields are more affected by the I/S ratio than the enzyme load. As has previously been discussed it is important to find the lowest I/S ratio where the methane yield remains high, in order to increase the total methane production. During this work the highest methane yield (284.37 mL CH_4_/gVS) was achieved at a low I/S ratio, equal to 0.7, with the addition of 13 FPU/g resulting in a total production of 4.7 L CH_4_/L.

## 4. Conclusions

The ability of utilization of dried sweet sorghum stalks as raw material for anaerobic digestion was demonstrated. One-step enzymatic treatment of stalks resulted in an increase of the methane production yield compared to thermal treatment which resulted in slight decrease. Finally, the combined effect of enzyme load and I/S ratio was evaluated resulting in higher yields and total methane production.

## Figures and Tables

**Table 1:** Codded and actual values of the experimental design.

Treatment	Coding values (*X*_1_ = enzyme load, *X*_2_ = I/S ratio)		Actual values (*X*_1_ = enzyme load, *X*_2_ = I/S ratio)	
	*X*_1_	*X*_2_	*X*_1_	*X*_2_
1	−1	−1	3	0.7
2	1	−1	13	0.7
3	−1	1	3	3.3
4	1	1	13	3.3
5	−1.414	0	0.93	2
6	1.414	0	15.07	2
7	0	−1.414	8	0.16
8	0	1.414	8	3.83
9	0	0	8	2
10	0	0	8	2
11	0	0	8	2

**Table 2:** Experimental obtained and predicted methane yields.

Treatment	Met (methane yield, mL CH_4_/g VS) experimental		Met (methane yield, mL CH_4_/g VS) predicted
	A	B	
1	267.15	273.02	269.57
2	277.92	290.82	283.23
3	210.68	225.40	213.51
4	256.48	239.09	242.64
5	229.42	246.11	240.16
6	274.20	260.08	270.41
7	47.11	49.86	—
8	201.86	216.86	215.03
9	242.05	256.17	254.56
10	257.15	249.21	254.56
11	259.60	263.16	254.56
